# Dental Legislation

**Published:** 1875-10

**Authors:** 


### DENTAL REGISTER.

Vol. XXIX.] OCTOBER, 1875. [No. 10.

### DENTAL LEGISLATION.

*Extracts from the Annual Address of Dr. W. C. Barrett, President of the New York State Dental Society, held at Albany, June 30th, 1875.*

Whether, in the estimation of the world, Dentistry shall be classed as a liberal and learned profession, or shall degenerate into a mere handicraft trade, rests, in a great measure, with the dentists of to-day. What shall be its future, is a question well worthy the attention of every practitioner, who has a thought above the mere bread and butter aspect of the case. To him who is of the earth earthy, it matters little what his occupation be denominated, so only that it supplies his gross material wants. If he has no tastes or aspirations, other than those connected with mere bodily demands, it is little likely that he will care for the æsthetics of his business.

Dentistry has not yet reached adult years, and its definite place among the various callings of men has not been fully assigned it. Whether it shall be considered an independent profession, or a particularity of medicine;—whether the operative and mechanical departments, shall be but integral parts of a complete whole; or within each shall be relegated to distinct, and separate spheres, is yet a question, both sides of which have their advocates. But that it is the duty of every dentist who loves Dentistry, to do his utmost toward assigning it, its proper position, no one will deny. This is a labor worthy the efforts of the best, for he will thus be assisting to determine the proper states of an art, to which the world will be indebted for much of its general welfare and immunity from suffering. In its nonage, he is shaping the existence of that, which will exert a powerful influence upon the future of those, who, for coming ages, shall stand in the relation of dentist and patient. Just here, and now, a twist of the young shrub, may result in a future monstrosity. We ought then, in what we do, to have a care that passion or prejudice do not dominate us.

There are those, who insist that dentists are medical men, practicing a specialty. Have they sufficiently considered the question, in all its bearings? In order to the pursuing a specialty, we necessarily presuppose an acquaintance with general practice, and a subsequent divergence from the consideration of all parts of a science alike, and the exclusive devotion to a particular branch. The greater includes the less, but a part cannot comprise the whole. Medicine is a science, and he who only studies a subdivision of it, cannot be said to be versed in medical science. He may thoroughly master some portion of it, but doing this, he is only partially a medical man. If he would take rank as a physician, he must first master general medical science, and not one of its branches. An oculist may be pursuing a specialty, but not a specialty *in medicine*, till he first become a general practitioner; that is, until he graduates in medicine, he cannot practice, either generally or specially in that profession. How can we avoid the conclusion, that an attempt to bolster ourselves up by clinging to another profession, is an error on our part, and that the resentment, shown by the medical profession, toward our rather impertinent claims, is but the exhibition of proper spirit on their part. That we are nearly akin, they cannot, and will not attempt to deny; but that we, without any such thorough, and radical training, as they themselves have submitted to, should be admitted within the precincts, which for ages has been so jealously guarded, we may not expect. There is but one door through which the outsider may enter thė ranks of medicine, and he who attempts to climb up by any other way, will be justly denounced as a thief, and a robber. Our course in the past has, in many instances, been ill-considered. We have attempted to gain recognition, by claiming something more than mere consanguinity with medicine! We have endeavored to shine by a borrowed light! With our separate schools, our distinct literature, and our peculiar degrees, which medical colleges are not competent to grant, we have yet desired to shelter ourselves under another organization. As if admitting our inability to stand alone, we have asked that we might lean against the structure of our neighbor.

There is a higher ground, a more noble position-which we may, and should occupy,—that of a separate, and distinct learned profession. We have schools, and a literture, equal to any, with a practice demanding special literary and professional training. These are subjects, closely connected with our practice, that are vital to the interests of mankind, and which can alone be properly studied, and their mysteries solved by those who have had the advantage of our professional training,—questions in science, which we alone are competent to answer. Then, with our colleges specially devoted to the training of those who would master the mysteries of our guild, with a separate literature, and a distinct mission in the broad field of science, what hinders one taking the position which rightfully belongs to us—that of a separate and distinct profession? Nothing! except a full understanding, and proper appreciation of our duties, and a thorough organization; for a simple agglomeration of particles, does not constitute a work of art.

It is urged with truth, that we shall take precisely the rank to which we, as dentists and scientific men, are worthily entitled. True enough perhaps, but *when* shall that position be accorded us. Harvy and Jenner were for many years denounced as quacks, and old established professions are not, usually, quick to acknowledge new truths. It is our duty to substantiate our claims to a portion of the field of services, not arrogantly, but modestly, and firmly. We possess all the elements of a profession, but so long as we are but an unorganized mass, with no responsible head, we cannot write our true professional influence. We have schools, a literature, learned men, enthusiastic students in science, whose form is not circumscribed by the boundaries of state or county, but a mob, even of officers, is not an army. For proper and effective organization, there must be some central heart, from which shall radiate, and toward which shall convey, all the life currents, so necessary to the support of an organism. Heretofore, the efforts for the advancement of Dentistry, have been individual and desultory. While each was anxious that his chosen profession should secure the respect of others, there has not been a necessary harmony of action. All have desired recognition from our near kindred, medicine. But there has been no unit for their acknowledgement; there has been no responsible head, to which the avowal might be made. Themselves thoroughly organized in schools, with clearly defined lines of demarcation, they have hesitated to give the hand of fellowship to an irresponsible, disorganised aggregation, however respectable otherwise. Having a settled habitation, they are distrustful of the dwellers in tents. There are many denied courtesies for which we must look to Law, and we wish even to hold up our heads in the presence of theology. But if we except the profession in a very few of the states, we have no settled existence. We are of such a recent growth, that our proper position has not been determined. Medicine dates from remote ages.

Hippocrates was a physician, and there have been doctors, ever since men were subject to disease. Law dates from the time when, for mutual protection, men first banded themselves together, and sought the services of some Moses or Solon, to lay down maxims by which their inter-communications might be governed. Theology is the outgrowth of natural principle in man, which continually looks up, toward something not subject to the infirmities of our weak natures; the natural religion of the soul, ever seeking for something worthy its adoration. Medicine may degenerate into Charlatanism, and Superstition: Law become the fawning sycophant, or the shyster; and theology be degraded into priest-craft. Yet in spite of all, whenever men have become an aggregation, there has been law to define social relation, medicine to heal bodily infirmities, and a priesthood to stand, as far as might be, a connecting link, between man and that mysterious Power, which in some form, all nations have worshipped. No people so savage, but has had its professors of Law, Medicine, and Divinity, from the brutal debased African tribe, or the wild American Indian Nation, whose superstition clothes their Medicine man, or Prophet, with the knowledge, and power, and influence of all others up to those races, highest in the scale of civilization, who have made such proficiency in scientific knowledge, that scarce does our life time suffice to master the literature of either. But no where, is either reckoned a profession, unless there be established some barrier, clearly marking its confines, and within which the ambitious youth is not admitted without a fully demonstrated fitness. Among the Indian tribes, it is required that the candidate for that office of combined Physician, Judge, and Priest, should have been devoted to that special purpose from infancy. Modern medicine demands the evidence of proper scholastic and professional training, in the form of a well earned diploma, before the neophyte is admitted to any kind of fellowship, or place in the ranks of his chosen profession. Law demands the examination of a candidate, under the responsible authority of a properly constituted court, and even when once admitted within the sacred precincts, the proper officers are armed with authority to thrust him out, on proof of subsequent unfitness. Every country minister must be inducted into his place, with due formality, and after close scrutiny, not only into his literary and professional, but moral qualifications. He must *believe* according to an established formula, and even his conscience, is placed under surveillance. It is only ourselves, who, claiming to be a profession, have established no line of demarcation within which a man is orthodox, and beyond which he must be professionally damned. Every man, woman or child, who mounting the housetop, proclaims himself or herself a dentist, is to a certain extent fellowshiped, and to him we all, high and low, extend the professional designation "Doctor." There is no absolute criterion by which those who claim a place in the ranks are to be judged. If we are indeed a specialty in medicine, it must be found in the diploma of Doctor of Medicine. If we are a profession by ourselves, there should be some recognized, legally accepted diploma, that shall determine a dentist's status. All our colleges should be empowered to grant but that one diploma, in course, and that should be the rule of measurement of a man's acquirements. Certain it is that some, without that evidence of scholastic training, will be better qualified for practice than others who have gone through the curriculum of the schools; but inasmuch as some standard by which to judge men must be erected, these must, until they have demonstrated their fitness before the properly constituted authorities, be classed with other outsiders. But instead of this uniform and recognized degree, we have the painful spectable of one of the leaders in dental reform, one of our oldest and most honored universities, of whom we had hoped great things, setting up a standard of its own, and confusiug the scientific world, by establishing a hitherto unknown degree. Instead of assisting in the thorough organization of our profession, which was the ostensible purpose in commencing their dental school, this honored seat of learning has but added to the already too great demoralization. The great desideratum among us is uniformity, and we are becoming more confounded.

If we would really become a profession, these things must not be. Thorough organization must establish the boundaries of dentistry, and cut off all communication with outside dental barbarians. To be continued.

